# Transgenic and gene knockout mice in gastric cancer research

**DOI:** 10.18632/oncotarget.12467

**Published:** 2016-10-04

**Authors:** Yannan Jiang, Yingyan Yu

**Affiliations:** ^1^ Department of Surgery of Ruijin Hospital and Shanghai Institute of Digestive Surgery, Shanghai Key Laboratory for Gastric Neoplasms, Shanghai Jiao Tong University School of Medicine, Shanghai, China

**Keywords:** mouse models, gastric cancer, histology

## Abstract

Mouse models are useful tool for carcinogenic study. They will greatly enrich the understanding of pathogenesis and molecular mechanisms for gastric cancer. However, only few of mice could develop gastric cancer spontaneously. With the development and improvement of gene transfer technology, investigators created a variety of transgenic and knockout/knockin mouse models of gastric cancer, such as *INS-GAS* mice and gastrin knockout mice. Combined with *helicobacter* infection and carcinogens treatment, these transgenic/knockout/knockin mice developed precancerous or cancerous lesions, which are proper for gene function study or experimental therapy. Here we review the progression of genetically engineered mouse models on gastric cancer research, and emphasize the effects of chemical carcinogens or infectious factors on carcinogenesis of genetically modified mouse. We also emphasize the histological examination on mouse stomach. We expect to provide researchers with some inspirations on this field.

## INTRODUCTION

Gastric cancer is the third leading cause of cancer death, and the fifth most common malignancy in the world [1]. The underlying pathogenesis of gastric cancer still remains unclear. It is important to develop an authentic animal model to imitate process of human gastric carcinogenesis. Genomic sequencing studies demonstrated that the protein-coding genes of both mice and humans shown high similarity [2]. Since mice disclosed the superiority on short inter-generation interval, high reproducibility as well as similar genetic background and formula experimental protocols, compared to other species, they have attracted considerable attentions as useful models to uncover the mechanisms of diseases and cancer, and to use for preventive and therapeutic interventions [3].

However, mice rarely develop gastric cancer spontaneously. To get a cancerous phenotype, researchers must feed them with *helicobacter* or chemical carcinogens. Along with the development of genomic manipulation, peoples could quickly create genetically engineered mice. The appearance of gene modified mouse models enabled us to explore the association of unique genotype with the specialized phenotypes. The creation of genetically engineered mice is mainly based on gene transfer technologies. In early stage, researchers studied the association of disease with mouse genetics by means of spontaneous or induced mutation models [4-6]. The technology of gene transfer is also called ‘‘reverse genetic’’ approach. In this approach, an identified gene which involved in human disease is selected as a target of gene manipulation in mice [3]. This approach is proper for uncovering the relevance of unique cell types or genetic pathways in pathogenesis of some diseases. The mouse with exogenous genome sequences is called transgenic mice, and the mouse with lost or altered endogenous gene is called knockout/knockin mouse. If the expression of targeted gene is controlled in specific time or organs/cells, we call it conditional transgenic or knockout/knockin mice. Generally, transgenic mouse was created to investigate the consequences of gene over-expression, and the knockout/knockin mouse was used to study the impacts of gene low-expression or mutation.

## CONSTRUCTION OF GENETICALLY ENGINEERED MICE

Typically, there are two basic approaches to produce genetically engineered mice, one is carried out through microinjection of DNA into the pronucleus of zygotes, the other is based on manipulation at embryonic stem cells. Generation of traditional transgenic mice depends on pronuclear microinjection of DNA, while generation of traditional knockout mice depends on embryonic stem cells and homologous recombination techniques, which get a higher success compared to other strategies. However, the complete process requires more than a year to generate a genetically modified mouse. The technology of site-specific nucleases provided a new choice for rapid generation of transgenic models, including zinc finger nucleases (ZFNs), transcription activator-like effector nucleases (TALENs), and clustered regularly interspaced short palindromic repeat (CRISPR) systems. Compared to ZFNs and TALENs, CRISPR-mediated genome engineering is easy and efficient. Scientists could directly inject CRISPR systems into zygotes and haploid ES cells instead of ES cells to shorten the breeding time. In this paper, we introduce the generation of transgenic mice, ES cells targeted knockout mice and the latest CRISPR-Cas knockout mice briefly.

Transgenic mice are strains of mice, which express exogenous genes or DNA sequences. One of the most commonly used methods to create transgenic mice is DNA pronucleus microinjection, by which, exogenous DNA is injected into one of the fertilized pronucleus eggs. Exogenous DNA will be integrated into the genome of mouse fertilized eggs and pass on to offsprings stably. Fertilized eggs usually come from C57BL/6 mice or FVB mice. The former stain is the source of published mouse genome sequencing. The latter stain has the advantage of easy pronucleus injection due to its big and clear pronucleus. By different DNA sequences injection, researchers will get transgenic mice with expression of human genes, microRNAs, small interfering RNAs or BAC/YAC gene sequences.

**Figure 1:**
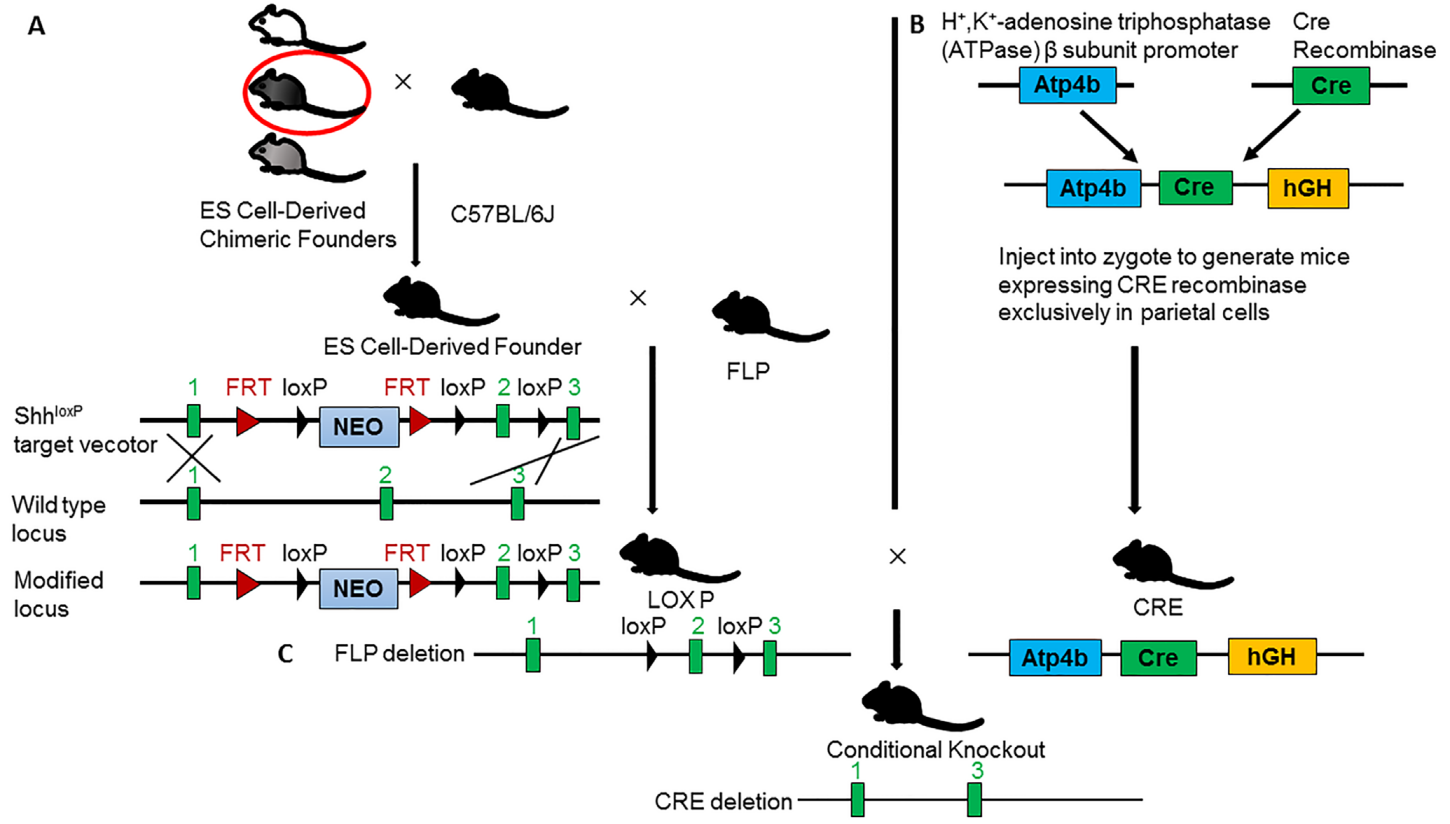
The flowchart of creating conditional knockout mice with specific ablation of *Shh* in gastric parietal cells **A.** Crossing high degree chimeric founders with C57BL/ 6J mice. Targeted ES cells are transferred to the blastocoel cavities of 3.5 day blastocyst embryos, and then, the embryos are transferred to surrogate mothers to complete gestation. The offspring are chimeric mice with two sets of genome. The chimeric degree is judged by coat color. Since the ES cells are come from black mice, while blastocyst embryos are from white mice, so, the greater the share of black, the higher degree of chimeric. **B.** Deletion of selected gene Neo. The FLP can recognize *FRT* sequence and remove the fragment between two *FRT*. Crossing the ES cell-derived founder with FLP mice to obtain F1 heterozygous mice (*Shh*) without *Neo* gene. Further crossing F1 heterozygous mouse to generate F2 homozygous (*Shh*) mice, which carry loxP-Shh-loxP in chromosomes. **C.** Creation of conditional knockout of *Shh* in gastric parietal cells. Since CRE recombinase is specifically expressed under the control of Atp4b promoter in gastric parietal cells, researchers should cross *Shh*mice with *Atp4b-Cre* mice, and get the conditional knockout mice with exon2 deletion of *Shh* gene in gastric parietal cells (*Shh; Atp4b-Cre*). In *Shh* conditional knockout mice, *Shh* is not expressed in gastric parietal cells, but normally expressed in other cells.

**Figure 2:**
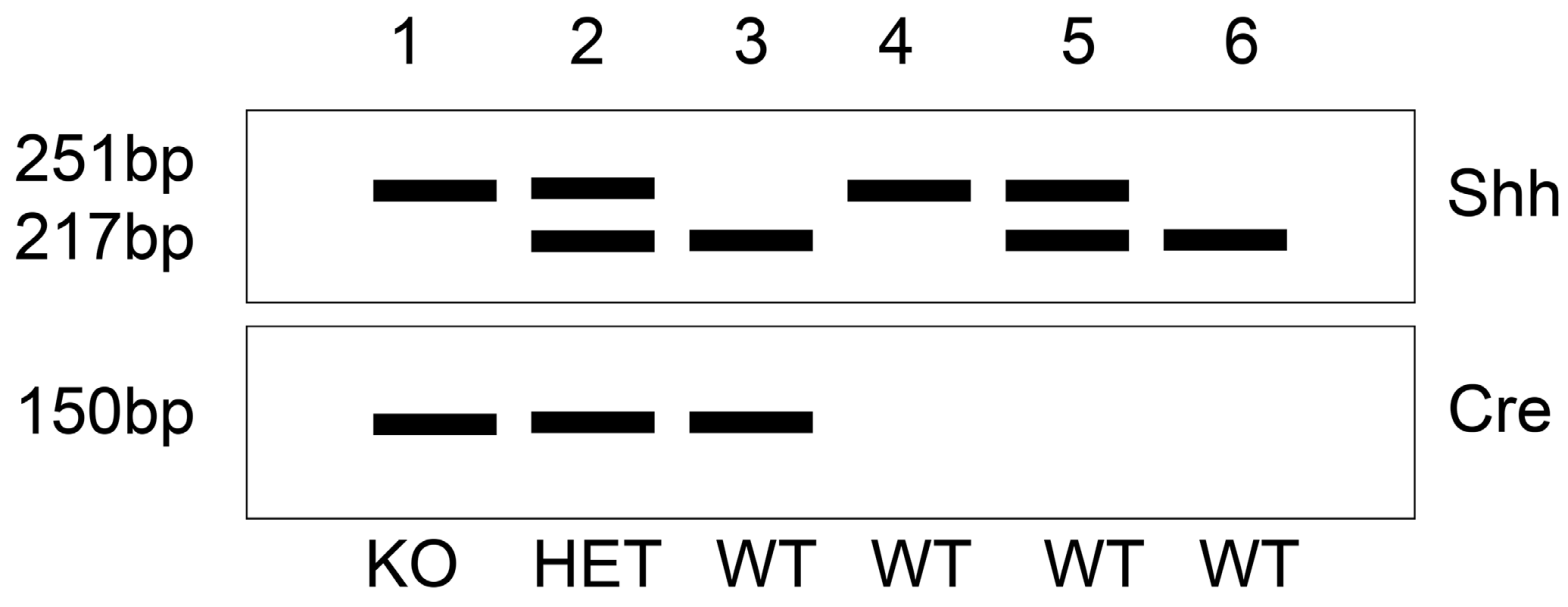
Schematic figure of genotyping for evaluating conditional knockout mice (*Shh; Atp4b-Cre*) Evaluation of mouse genotyping is based on polymerase chain reaction (PCR). Primers are designed to modified *Shh* gene and *Cre* recombinase. The size of PCR product of floxed *Shh* is 251bp, while the wild type is 217bp. Heterozygous genotype show two products of both 251bp and 217bp. The size of *Cre* product is located at 150bp. The samples DNA from mouse 1, 2 and 3 are *Cre* positive. Mouse 1 represents homozygous knockout mouse (KO). Mouse 2 is heterozygous genotype (HET), and mouse 3, 4, 5 and 6 are wild type mice (WT).

Gene target mice are divided into complete knockout mice, conditional knockout mice and gene knockin mice. Complete knockout mice are mice with deletion of target gene in whole tissues and cells of body. These mice can be used to study the function of target gene in the physiology and pathology of the whole body. Since complete knockout mice disclosed higher embryonic lethal rate, the utility of complete knockout mice is limited. To avoid the problem of embryonic death, another kind of knockout mice, conditional or inducible knockout mice are developed. These mice are generated using the LoxP-Cre system. Two LoxP loci are inserted into one or several important exons of targeted gene to produce floxed mice. These mice express targeted gene normally when its genotype is wild type. After crossing floxed mice with Cre mice, the floxed exons will be deleted, and resulted in knockout of targeted gene. The exogenous Cre expression is depended on control of proper promoter in Cre mice. That is, different promoters drive Cre expressed in different tissues and cells. For example, Onoyama and coworkers generated mice with liver-specific null mutations of *Fbxw7* by means of two different Cre-loxP systems (*Mx1*-Cre and Alb-Cre) [7]. Cre expression occurs in Alb-Cre mice embryos and maintains lifelong, while *Mx1* gene promoter was activated only after injection of poly(I)-poly(C) into mice. Up-to-date, there are several kinds of Cre mice expressing CRE recombinase in stomach, including Atp4b-Cre (express CRE in parietal cells), Capn8-Cre (express CRE in glandular pit cells), Pgc-Cre (express CRE in chief cells), K19-Cre (express CRE in glandular isthmus cells), Villin-Cre (express CRE in glandular progenitor cells). The details of Cre mice and floxed mice can be viewed in MGI website (http://www.informatics.jax.org/). The following flowchart is an example of creation of Shh (Sonic Hedgehog) conditional knockout mice in gastric parietal cells (Figure 1). The method of genotyping detection is summarized in (Figure 2) [8]. Based on literature, the mouse models with conditionally targeted gene knocked out at stomach are listed in Table 1.

CRISPR-Cas is a new technology that has emerged recently, and obtained widespread attention. In CRISPR-Cas system, a sequence-specific guide RNA is used to guide nuclease to create a nick on targeted location. A new sequence is inserted by homologous recombination. CRPSPR-Cas system consists of clustered regularly interspaced palindromic repeats (CRISPR) and the CRISPR-associated proteins (Cas), which have the structure of nucleic acid enzyme activity. The CRISPR-Cas was divided into several subtypes. Each CRISPR-Cas has its unique features in biochemistry and molecular genetics. CRISPR-Cas9 is widely used in generation of genetically engineered mouse [9].

**Table 1:** The targeted genes are conditionally knocked out in stomach as well as other organs on mouse models*

Model name	Stomach	Other organs	References
Foxa3-Cre	Glandular stomach cells	Liver, pancreas, intestine, ovary, testis, heart and adipose tissue	[10-14]
Capn8-Cre	Gastric pit cells	Liver and skin	[15]
Pdx1-Cre	Endocrine cells of gastric antrum (such as gastrin, somatostatin and serotonin)	Endocrine cells of duodenum and pancreatic islet cells	[16-18]
Villin-Cre	Gastric progenitor cells	Progenitor cells of intestine and kidney proximal tubules	[13, 17-19]
Edn2-iCre	Stomach cells	Cells of periovulatory ovary and intestine	[20]
Tff1-CreERT2	Epithelium of the glandular stomach	ND	[21]
Atp4b-Cre	Parietal cell	N	[22]
Lgr5-CreERT2	Base cells of glands in gastric pylorus and corpus region	Base cells of intestine and hair follicle	[23]
Tff2-CreERT2	Parietal cell, mucous neck cell and chief cells	ND	[24]
H(+), K(+)-ATPase β-subunit promoter	Parietal cell	N	[25]

* N, No; ND, Not detectable

## PATHOGENIC STUDY OF TRANSGENIC/KNOCKOUT/KNOCKIN MICE

### *INS-GAS* mice

Gastrin is a crucial hormone produced by antrum G cells of gastric mucosa, and responsible for gastric acid secretion and oxyntic cells differentiation. Insulin-gastrin (*INS-GAS*, FVB/N background) transgenic mice were originally created to examine the possible role of gastrin in regulating islet differentiation. The *INS-GAS* transgenic mouse contains two exons of the human gastrin gene, which encode progastrin precursor under control of insulin promoter [26]. *INS-GAS* mice have also been used to investigate the effect of gastrin on gastric cancer development [27]. *INS-GAS* mice showed increased maximal gastric acid secretion and increased parietal cell number in early stage, but gradually changed into hypochlorhydria and decreased parietal cell number later (five months later). On the age of 20 months, *INS-GAS* mice exhibited metaplasia, dysplasia and gastric cancer. *H felis* infection could accelerate (≤8 months) these lesions [28]. The tumorigenesis of *INS-GAS* mice revealed differences in strain and gender. The mice with FVB/N background is more susceptible to gastric cancer, while C57/BL6 background mice is not susceptible to gastric cancer, and only develop hyperplasia and low-grade dysplasia [29]. In addition, male mouse is more susceptible to gastric cancer compared to female mouse [30]. *INS-GAS* mouse with *helicobacter* infection has been widely used as a model of intestinal type gastric cancer. Scientists use this mouse model to study the change of gene expression induced by *H pylori* infection at the high gastrin status and found a group of differential expressed genes [31]. Lofgren compared INS-GAS mice raised in germ-free (GF) and SPF with intestinal flora conditions and found that intestinal flora promoted gastric lesions [32]. Lertpiriyapong compared INS-GAS mice raised in germ-free, altered Schaedler's flora and intestinal flora conditions, and then infected by *H Pylori.* They found that mice at altered Schaedler's flora and intestinal flora conditions could develop severer gastric lesions. They believe that colonization efficiency of commensals is important [33]. Whary infect INS-GAS mice with Heligmosomoidespolygyrus and *H Pylori*, and found that coinfection of Heligmosomoidespolygyrus with *H Pylori* could decrease *H Pylori* -induced atrophy and dysplasia [34].

### *Gastrin* knockout mice

Gastrin knockout mice (C57BL/6 strain, *GAS ^-/-^*) were originally generated by Koh and Friis separately through gene targeting [35, 36]. Mutant mice were viable and fertile, but secretion of gastric acid was abolished, leading to a marked change in gastric architecture, with decreased parietal cell number and enterochromaffin-like (ECL) cells, and increased mucous neck cells. There were no differences in the proliferation labeling index of the stomach between gastrin-deficient mice and wild type littermates. Some researchers found that *GAS ^-/-^*mice of 129/Sv strain kept in nonbarrier rooms led to bacterial overgrowth and elevated numbers of parietal cell, G cell and inflammation [37]. At the age of 12 months, these mice developed stomach tumors [38]. *GAS ^-/-^*mice are proper for studying biological functions of gastrin-regulated gene (*Tff1* and *Tff2*) in maintenance and repair of gastric mucosa [39, 40]. INS-GAS transgenic mice and GAS knockout mice are often used together in one research to study the role of gastrin in gastric carcinogenesis, but the results were not always concordant. The different results may be due to the different backgrounds of mice strain and different location on stomach, for example the corpus or antrum. It seemed that gastrin promote cancer development in corpus but not antrum [29].

### *Tff1* knockout mice

Trefoil factor 1 (*TFF1*, formerly known as *pS2*) is a tumor suppressor gene that encodes a peptide belonging to the trefoil factor family. TFF1 expression is frequently lost in gastric carcinomas [41]. As early as in 1996, Lefebvre and colleagues generated *Tff1* knockout (*Tff1^-/-^*) mice to explore biological function of this gene [42]. Homozygous mutant mice of *Tff1 (Tff1^-/-^)* develop antropyloric adenoma, and even multifocal carcinomas, consistent with increased inflammatory scores [43]. They identified NF-κB to be a network hub that is activated in *Tff1* knockout mice through mRNA microarray analysis on antral samples from *Tff1^-/-^* mice and wild type mice. Another group reported MNU-driven tumorigenesis on *Tff1^+/-^* mice. They found increased antral proliferation and progenitor cell number at age of 18 weeks, and increased malignant tumors in heterozygous mice (*Tff1^+/-^*), compared to wild type mice. In addition, they found that mRNA expression of *Tff1* was almost lost in heterozygous mice (*Tff1^+/-^*) [44]. The *Tff1^+/-^* mice are applied for studies on gene heterozygocity and transcript regulation.

### *IL-1β* transgenic mice

Polymorphisms of interleukin-1beta (*IL-1β*) involved in enhancing production of *IL-1β* are associated with an increase risk for both hypochlorhydria induced by *H. pylori* and gastric cancer [45]. Tu *et al.* generated *IL-1β* transgenic mice by using an H/K-ATPase/hIL-β transgenic mouse that express human *IL-1β* specifically in stomach. The *IL-1β* transgenic mice spontaneously develop chronic gastritis, metaplasia and high-grade dysplasia/carcinoma. In the setting of *H felis* infection, these mice show accelerated development of gastric inflammation and carcinoma compared to control mice [46]. In human beings, *IL-1β* synergizes with *H pylori* and increases the risk of gastric cancer. Moreover, overexpression of *IL-1β* in transgenic mice could recruit the accumulation of myeloid-derived suppressor cells (MDSCs) through NF-κB signal pathway. MDSCs are important for carcinogenesis in the early stage of gastric carcinogenesis. *IL-1β* transgenic mouse is widely used for testing the efficacy of anti- *IL-1β* therapies in cancer prevention and function of MDSCs in tumor microenvironment [47]. IL-1β knockout mice were also created and treated with *H pylori*. These mice exhibited decreased recruitment of macrophages and neutrophils by *H pylori* infection and reduced activation of NF-kB [48].

### *K-ras* transgenic/knockin mice

*K-ras* gene mutations have been found in about ~6% or ~18% of diffuse type or intestinal type of gastric cancer, respectively [49]. The effect of RAS protein is complex with either positive or negative effects on cell growth, differentiation and death [50]. Brembeck and coworkers created *K-ras* transgenic mice under control of cytokeratin19 (*K19*) promoter [51]. *K19* was expressed in an epithelial-specific pattern, restricted to ductal epithelial cells in the pancreas, surface colonocytes, small intestinal villi and gastric isthmus cells. Despite the findings in pancreas, *K-ras* transgenic mice exhibited concomitant parietal cell decrease and mucous neck cell hyperplasia (3-6 months age) [52]. The *CK19^CreERT^; LSL-Kras^G12D^* mouse is a kind of *K-ras* mutation (G12D) mouse, which showed metaplasia, foveolar hyperplasia, reduced presence of parietal cells and a deeper proliferative zone in the fundus of stomach at the age of 4-6 months after tamoxifen administration [53]. Matkar and colleagues found that systemic activation of *K-ras* leads to rapid changes in gastric cellular homeostasis and causes hyperplasia of the forestomach and the glandular stomach, depletion of parietal cells, accompanied by upregulation of inflammatory response factor (*COX-2*), stem cell marker (*Dcamkl1, CD44*) and activated MAPK pathway. Therefore, systemic *K-ras^G12D^* activated mice function as a tool for studying the early molecular events of gastric carcinogenesis [54].

### *Apc and Wnt1* transgenic mice

Adenomatous polyposis coli (*APC*) is a key tumor suppressor gene that acts as an antagonist of Wnt signaling pathway by maintaining cytoplasmic levels of β-catenin [55]. About 88% patients with familial adenomatous polyposis caused by APC germline mutations develop to fundic gland polyps, while fundic glandular polyp may transform to adenocarcinoma [56]. However, *APC* gene mutation is frequently found in gastric adenomas, but rarely in gastric cancer. For instance, one *Apc* mutation (*Apc*1638) led to gastric dysplasia and polyposis in antrum and pyloric junction [57, 58]. A transgenic mouse carrying *Apc* gene mutations infected by *H felis* developed less gastritis, less epithelial proliferation and inflammation, compared to wild type mice. It means the immune and inflammatory response of *Apc* gene mutations were not serious [59]. To explore Wnt pathway on gastric carcinogenesis, Oshima and coworkers constructed *K19-Wnt1* transgenic mice, which express Wnt1 in gastric mucosa. They crossed *K19-Wnt1* mice with *K19-C2mE* transgenic mice to investigate the role of *Wnt* and *PGE2* on gastric carcinogenesis [60]. The phenotype of *K19-C2mE* mice is overexpressed *COX-2* and microsomal prostaglandin E synthase-1 (*mPGES-1*) in gastric epithelium, resulted in increased metaplasia, hyperplasia and tumors in the glandular stomach with heavy macrophage infiltrations [61]. In addition, Akaboshi and coworkers used Hmga1-knockin mice crossed with *K19-Wnt/C2mE* mice and proved that Hmga1 is involved in gastric carcinogenesis *via* *Wnt/β-catenin* pathway [62].

### *p53* knockout mice

Mutations of *p53* gene constitute one of the most frequent molecular events in human cancers. As we know, *p53* knockout mice are highly susceptible to spontaneous tumorigenesis at age of 6 months [63, 64]. Since homozygous knockout animals could not be maintained long term, most of experiments of *p53* knockout mice are based on heterozygous knockout mice (*p53^+/-^*) rather than homozygous knockout mice (*p53^-/-^*). The incidence of invasive adenocarcinomas in the stomach of *p53^+/-^*mice was significantly higher than that in WT mice [65]. Infection of *H felis* for both WT and *p53^+/-^*mice was disclosed active chronic inflammation and marked mucosal hyperplasia at the age of 6 months [66]. There is a synergistic action between infection with *H felis* and *p53* deficiency in the accumulation of mutations in stomach [67]. However, another research revealed that *H pylori* (24w after infection) did not result in significant difference on the level of gastric epithelial apoptosis and proliferation between *p53 ^+/-^* mice and WT mice [68]. In order to explore the synergistic effect of two or more different genes, Shimada and coworkers created double conditional knockout (DCKO) mice by crossing *Atp4b-Cre* mice with *Cdh1^fl/fl^* and *p53^fl/f l^*mice, to examine the synergistic effect of E-cadherin loss and *p53* loss on stomach. The DCKO mice exhibited phenotypes of loss of cell polarity for parietal cells and proton pump-negative atypical foci, ultimately progressed to intramucosal cancer (9 months) and invasive cancer (12 months) [69]. Moreover, Park *and coworkers* crossed Pdx-1-Cre mice with *Smad4 ^fl/fl^* *p53 ^fl/fl^ Cdh1 ^fl/+^* and found that *E-cadherin* loss and *Smad4* loss cooperate with *p53* loss could promote the development of gastric cancer [17, 18].

### *Klf4* knockout mice

The Kruppel-like factor 4 (*KLF4*) is a zinc finger transcription factor that regulate numerous biological processes including proliferation, differentiation, development and apoptosis. KLF4 expression is found primarily in post mitotic phase and terminal-differentiated epithelial cells such as skin, lungs, and gastrointestinal tract [70,71]. Loss of *KLF4* expression was significantly associated with poor survival in gastric cancer [72]. Li and coworkers created Klf4 conditional knockout mouse models by crossing *Villin-cre* transgenic mice and *Klf4 ^fl/fl^* mice [13]. Villin is an actin-bundling protein located in the apical brush border of absorptive epithelium of intestine, as well as gastric progenitor cell (GPC). Villin positive GPCs are quiescent in unstimulated stomach with multilineage potential. By inflammation stimulation, these cells could undergo symmetric or asymmetric division and gradually replace pyloric glands [73]. The antrum mucosa cells revealed disturbed *Klf4* expression in *Klf4* knockout mice. At the age of 35 to 50 weeks, these *Villin-cre*-*Klf4* knockout mice developed preneoplasia in antrum, and 29% of them progressed to gastric cancer at 80 weeks. Chemical reagent MNU could accelerate tumor formation at 35 to 50 weeks of age.

## HISTOLOGICAL FEATURES APPEARED ON MOUSE STOMACH

**Figure 3:**
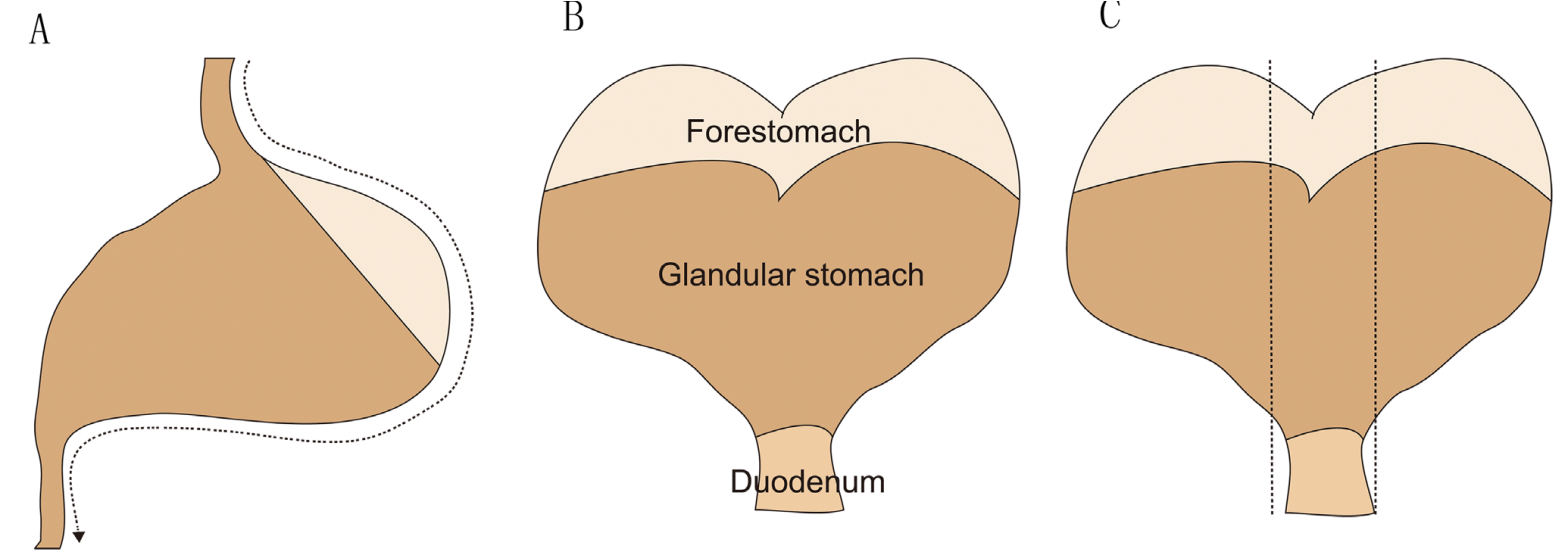
The schematic figure of dissecting method for mouse stomach **A.** The first incision is cut along the greater curvature from esophagus through proximal duodenum. **B.** After opening the stomach, the stomach is laid flat on a cutting board. **C.** Two or three linear strips are cut from the lesser curvature including squamocolumnar junction, corpus, antrum and pyloru and proximal duodenum.

In order to evaluate experimental effects, researchers need to observe gastric histology on different stages by necropsy. The adult mouse's stomach is located in the left cranial part of the abdominal cavity. The forestomach forms the left half, the glandular stomach forms the right half of the stomach. The wall has three layers: the mucosa with submucosa, the muscularis, and the serosa. In the forestomach, the mucosa is lined by stratified squamous epithelium covered by cornified tissue layer. It borders on that of the glandular stomach by a mucosal fold called the limiting ridge. The mucosa of the glandular stomach is lined by single columnar epithelium forming deep foveolae. The main part of the glandular stomach is fundic glands containing surface mucous cells in the pit zone, granular eosinophilic parietal cells (oxyntic cells) secreting hydrochloric acid and mucin-secreting non-chief cells lining the neck, and basophilic chief cells (zymogenic cells) producing prepepsin and located at the base [74, 75].

(Figure 3) indicates the dissecting method of mouse stomach. The first incision is cut along the greater curvature from esophagus through proximal duodenum. After opening the stomach, the stomach is laid flat on a cutting board. Two or three linear strips are cut from the lesser curvature including squamocolumnar junction, corpus, antrum and pyloru and proximal duodenum. In general, stomach tissues are collected and fixed in 4% formalin in PBS overnight, and processed for standard paraffin histology. Slides are stained by hematoxylin-eosin reagent or mucin stain and scored for pathology on a scale of 0 to 4. Regarding to *H pylori*-associated gastric histology, there are six parameters for histological evaluation, such as inflammation, epithelial defect, oxyntic atrophy, hyperplasia, pseudopyloric metaplasia, and dysplasia or tumor. Sometimes, some parameters are not appeared on chemistry-induced lesions. Here, we present several crucial histological evaluation parameters: corpus and antral inflammation; corpus glandular atrophy; mucus metaplasia; dysplasia and carcinoma. Among them, mucus metaplasia and dysplasia belong to precancerous lesions. The corpus and antral inflammation is defined by sub-mucosal and mucosal presence of polymorphous nuclear and mononuclear cells, which is scored according to the extent of inflammatory cells. Infiltration: 0, no inflammatory cells; 1, inflammatory cells infiltration of the submucosa with or without infiltration at the very base of the mucosa; 2, inflammatory cells infiltration of the submucosa and the bottom half of the mucosa; 3, inflammatory cells infiltration of the submucosa and greater than 50% of the mucosa; 4, transmural infiltration of inflammatory cells. Corpus glandular atrophy is defined as loss of parietal and zymogenic cells. Corpus glandular atrophy is scored based on the estimated percentage of parietal cell and chief cell loss within the corpus: 0, no visible parietal cell and chief cell loss; 1, 25% parietal cell loss and 50% chief cell loss; 2, 50% parietal cell loss and greater than 75% chief cell loss; 3, 75% parietal cell loss and 100% chief cell loss; 4, greater than 75% parietal cell loss and no chief cells. Mucus metaplasia is defined as the ectopic presence of Alcian blue stained acidic mucin associated with the acquisition of an elongated antral glandular structure within corpus glands. Mucus metaplasia is scored based on the percentage of the corpus mucosa showing replacement of oxyntic glands with elongated Alcian-blue stained glands reminiscent of antral mucosa: 0, no visible mucus metaplasia; 1, small foci were present; 2, up to one-third of the corpus was affected; 3, two thirds of the corpus were affected; 4, greater than two thirds of the corpus were affected. Dysplasia is defined as disturbed or haphazard glandular arrangement, loss of vertical orientation, back-to-back associations without intervening stroma, branching and infolding glands. At the cellular level, dysplastic features include altered nuclear size, hyperpleomorphism, poorly defined cell junctions, loss of nuclear polarity, and hyperchromatin with increased nuclear-cytoplasmic ratio [76]. Dysplasia is scored based on the following criteria: 0, no visible dysplasia; 1, appearing aberrant crypt foci including distortion of normal columnar orientation, increased diameter, asymmetrical cell piling, and back-to-back forms. 2, there is glandular infolding, branching, and more advanced cellular atypia such as increased nuclear-cytoplasmic ratio. 3, cellular distortion with haphazard arrangements, the lesion developed to carcinoma *in situ*. 4, highly dysplastic glands invade into the submucosa or beyond, such as deeper layers, vessels and lymphatics [77]. Table 2 summarized the names of genetically engineered mouse models and observed lesions.

**Table 2:** Genetically engineered mouse models and observed lesions*

Model name	Time\Lesion	Metaplasia	Hyperplasia	Dysplasia	Carcinoma	Invasion	Metastasis	Cancer type	References
Tff1-/-	12 months	ND	Y	Y	Y	Y	N	/	[78-81]
gp130757F/F	3 months	Y	Y	Y	Y	Y	N	Intestinal	[21, 74, 82, 83]
Cdx2 transgenic	12 weeks	Y	Y	N	Y	N	N	Intestinal	[84-87]
INS-GAS	20 months	Y	ND	Y	Y	Y	N	Intestinal	[28, 30, 88-90]
ACT-GAS	20 months	ND	Y	ND	Y	N	N	N	[90, 91]
Gastrin-/-	12 months	Y	Y	Y	Y	N	N	N	[38, 92, 93]
Atp4a-/-	12 months	Y	Y	N	N	N	N	N	[94-96]
NHE2-/-	3 months	ND	Y	N	N	N	N	N	[97]
NHE4-/-	9 weeks	N	N	N	N	N	N	N	[98]
Kvlqt1-/-	3 months	ND	Y	N	N	N	N	N	[99]
H2R-/-	16 weeks	N	Y	N	N	N	N	N	[100-102]
HDC-/-	12 months	Y	Y	N	N	N	N	N	[103]
IQGAP1-/-	15 months	ND	Y	Y	N	N	N	N	[104]
Tgf β1-/-	20 days	Y	Y	N	N	N	N	N	[105]
Smad4-/-	9 months	ND	Y	Y	Y	Y	N	/	[18, 106-108]
Runx3-/-	8 months	ND	Y	Y	N	N	N	N	[109, 110]
Apc +/-	20 weeks	ND	Y	Y	Y	Y	N	/	[111]
MTH1-/-	18 months	ND	Y	Y	Y	N	N	Intestinal	[112]
K19-C2mE transgenic	45 weeks	Y	Y	Y	Y	N	N	/	[63, 64, 113]
Tsp-/-	3 weeks	Y	Y	N	N	N	N	N	[114]
Tgf α transgenic	6 weeks	ND	Y	Y	N	N	N	/	[115-118]
AhR transgenic	3 months	Y	Y	Y	Y	Y	N	Intestinal	[119, 120]
Klf4 -/-	35 weeks	ND	Y	Y	Y	N	N	/	[13, 14]
p27-/-	60 weeks	Y	Y	Y	Y	N	N	Intestinal	[121]
Car9-/-	4 weeks	ND	Y	N	N	N	N	N	[122, 123]
CEA SV40 transgenic	37 days	ND	ND	Y	Y	Y	N	Intestinal	[124,125]
H+/K+-ATPase β subunit SV40 transgenic	12 months	N	N	Y	Y	N	N	/	[126, 127]
H+/K+-ATPase β subunit-/-	20 months	ND	ND	Y	Y	Y	N	/	[128]
Shh-/-	18.5 day embryo	Y	Y	N	N	N	N	/	[129]
Occludin-/-	10 months	N	N	Y	N	N	N	/	[130]
ClC2-/- Cdh1-/-p53-/-	9 weeks 6 months	N Y	N Y	Y Y	N Y	N Y	N Y	/ Diffuse	[131] [17, 69]

* Y, Yes; N, No; ND, Not detectable

## SUMMARY

Mice are important experimental model for human gastric cancer study. Along with the findings of multiple mutations of crucial functional genes by whole genome sequencing, the biological functions of multiple genes should be verified on cell models as well as on genetically engineered mice model. Scientists could make conditional knockout mice by Cre mice that express CRE protein specifically in stomach. Scientists could make transgenic mice *via* specific promoter, such as K19 (epithelial cell specific) and H-K-ATPase (parietal cell specific). At present, there are no perfect promoters for gastric carcinogenesis study. New technologies such as CRISPR-Cas system are under study. Regarding to animal experiment, combination of chemical carcinogen and biological carcinogen could accelerate the tumorigenesis. In near future, more and more mouse models will be created. Although development of a new mouse model is time-consuming, it is valuable for gene function study. However, we must understand that there are some differences such as immune system or their anatomic organs between mouse model and human being. The histological scoring criteria are helpful to improve comparison of results between different laboratories. The right evaluation will assist scientific investigators and medical professionals in understanding and objectively scoring disease progression in mouse models.
